# Case Report: Bronchogenic Cyst in the Right Atrium of a Young Woman

**DOI:** 10.3389/fcvm.2022.915876

**Published:** 2022-05-27

**Authors:** Yuya Fukudome, Michinari Hieda, Shiho Masui, Taku Yokoyama, Shutaro Futami, Shohei Moriyama, Kei Irie, Mitsuhiro Fukata, Tomoki Ushijima, Akira Shiose, Koichi Akashi

**Affiliations:** ^1^Heart Center, Kyushu University Hospital, Fukuoka, Japan; ^2^Department of Medicine and Bio-systemic Science, Hematology, Oncology, and Cardiovascular Medicine, School of Medicine, Kyushu University Hospital, Fukuoka, Japan; ^3^Department of Cardiovascular Surgery, Kyushu University Hospital, Fukuoka, Japan

**Keywords:** bronchogenic cyst, cardiac mass, cardiac biopsy, cardiac MRI, imaging

## Abstract

A 31-year-old woman was referred to our hospital for evaluation of a cardiac mass in the right atrium. Cardiac magnetic resonance imaging indicated a cystic mass filled with fluid accumulation in the right atrium. The mass was identified as a cardiac cyst and was surgically removed. Pathological examination revealed an extremely rare bronchogenic cyst. Bronchogenic cysts are benign congenital abnormalities of primitive foregut origins that form in the mediastinum during embryonic development. There is unusual clinical dilemmas surrounding the treatment plan for cardiac surgery or biopsy of cardiac masses, especially in patients with rare cardiac cysts. The anatomical location of the cyst can be related to various clinical symptoms and complications. In cases of indeterminate cardiac cysts, direct cyst removal without prior biopsy is of utmost importance.

## Introduction

The incidence of primary cardiac tumors is approximately 0.02%, with three-quarters of cardiac tumors are benign. Among these benign cardiac tumors, cardiac myxoma is the most frequent. Other major types of benign cardiac tumors include fibromas, rhabdomyomas, papillomas, lipomas, papillary fibroelastomas, hemangiomas, and bronchogenic cysts (1).

Bronchogenic cysts in the atrial septum are extremely rare. Regarding the intracardiac bronchogenic cysts, they have been reported in only 22 cases, to date (2). They are benign congenital cysts of primitive foregut origin that form in the mediastinum during embryonic development (3–5). These bronchogenic cysts represent 6% to 15% of primary mediastinal masses (6), and may develop in the neck, spinal dura mater, sub-diaphragm, diaphragm (7), or retroperitoneal regions (8). The heart is derived from the mesoderm, and its development differs from that of the ectoderm-derived respiratory system. The embryonic cardiac primordium is close to the primordial bronchial tree and foregut. Therefore, it has been indicated that migration of the embryonic cardiac primordium to the cardiac muscle site during abnormal budding may be involved in cardiac cyst development (9). In addition, ectopic bronchogenic cysts are often misdiagnosed preoperatively, because they have no imaging features and have different clinical manifestations (10). Therefore, multimodality imaging is the most important approach to establish an accurate diagnosis.

## Case Presentation

A 31-year-old woman presented to a local physician 2 months before her referral with palpitations and shortness of breath. At the time, a paroxysmal atrial fibrillation was detected by a Holter-electrocardiogram examination. Thereafter, successful catheter ablation of paroxysmal atrial fibrillation was performed. Presence of a cardiac tumor in the right atrium was suspected during catheter ablation. Therefore, the patient was referred to our hospital for further evaluation.

On presentation to our hospital, the patient had no symptoms. The patient’s medical history included asthma, emergency cesarean section for pregnancy-induced hypertension, paroxysmal atrial fibrillation, gestational diabetes, and type 2 diabetes mellitus. The patient was managed with edoxaban (60 mg daily) for paroxysmal atrial fibrillation and dapagliflozin (5 mg), sitagliptin (50 mg), and metformin (500 mg) for type 2 diabetes mellitus. The patient had no other pertinent family medical history.

Her height, weight, body mass index, and body surface area were 157.8 cm, 64.6 kg, 25.94 kg/m^2^, and 1.66 m^2^, respectively. The patient had body temperature, 37.2°C; blood pressure, 130/88 mmHg; pulse rate, 108 bpm; and O_2_ saturation measured by pulse oximetry, 98% on room air. Normal heart and lung sounds were ausculated without any murmur. The abdomen was soft and tender. There was no edema in the lower legs. Her blood test results are shown in Table 1. A 12-lead electrocardiogram showed normal sinus rhythm. Chest radiography revealed a cardiothoracic ratio of 43.9% without pleural effusion or pulmonary congestion. Transthoracic echocardiography revealed normal left ventricular ejection fraction (71%) and diastolic function (E/e’: 9.0) without valvular diseases. Although we attempted to visualize the tumor in the right atrium using a subcostal approach, it was unclear due to obesity.

**TABLE 1:** Blood data on presentation in our hospital.

Measure	Data	Reference range
White blood cell count (10^3/^μL)	12.42	3.3–8.6
Red blood cell count (10^6/^μL)	5.69	3.86–4.92
Hemoglobin (g/dL)	11.4	11.6–14.8
Hematocrit (%)	38.8	35.1–44.4
Platelet count (10^3/^μL)	521	158–348
Fibrinogen (mg/dL)	250	200–400
Fibrinogen degradation product (μg/mL)	2.5	≦ 5.0
D-dimer (μg/mL)	0.5	≦ 1.0
PT-INR (INR)	1.17	0.90–1.10
Total protein (g/dL)	8.2	6.6–8.1
Albumin (g/dL)	4.9	4.1–5.1
Blood urea nitrogen (mg/dL)	13	8–20
Creatinine (mg/dL)	0.44	0.46–0.79
Urea acid (mg/dL)	2.8	2.6–5.5
Total-bilirubin (mg/dL)	0.5	0.4–1.5
Direct-bilirubin (mg/dL)	0.1	0.0–0.3
Aspartate aminotransferase (U/L)	24	13–30
Alanine aminotransaminase (U/L)	65	7–23
Lactic dehydrogenase (U/L)	167	124–222
Alkaline phosphatase (U/L)	58	38–113
Gamma-glutamyl transpeptidase (U/L)	55	9–32
Creatine kinase (U/L)	49	41–153
Glucose (mg/dL)	100	73–109
Total-cholesterol (mg/dL)	201	142–248
HDL-cholesterol (mg/dL)	43	48–103
LDL-cholesterol (mg/dL)	116	65–163
Triglyceride (mg/dL)	424	30–117
C-reactive protein (mg/dL)	0.08	≦ 0.14
Na (mmol/L)	138	138–145
K (mmol/L)	4.8	3.6–4.8
Cl (mmol/L)	101	101–108
IgG (mg/dL)	1124	861–1747
IgA (mg/dL)	602	93–393
IgM (mg/dL)	128	50–269
CH-50 (U/mL)	60.0	31.6–57.6
Hb A1c (%)	6.7	4.9–6.0
Troponin T (ng/mL)	0.004	≦ 0.014
Soluble interleukin-2 receptor (U/mL)	256	156.6–474.5
Brain natriuretic peptide (pg/mL)	4.5	0.0–18.4
Carcinoembryonic antigen (ng/mL)	0.7	≦ 3.2
Carbohydrate antigen 19–9 (U/mL)	9.1	≦ 37.0
Neuron-specific enolase (ng/mL)	14.3	≦ 15.1
Cytokeratin 19 Fragment (ng/mL)	1.4	≦ 3.5
Squamous cell carcinoma antigen (ng/mL)	1.2	0.6–2.3

**FIGURE 1:**
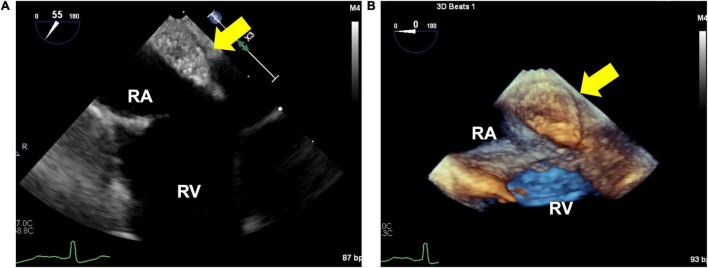
2-dimensional **(A)** and 3-dimensional **(B)** image of the isoechoic mass using transesophageal echocardiography. **(A)** 16×10×10 mm isoechoic mass. This image shows an isoechoic mass on the right interatrial septum near the inferior vena cava. **(B)** Mass is well-defined, has a smooth surface, and broadly adherent to the atrial.

**FIGURE 2:**
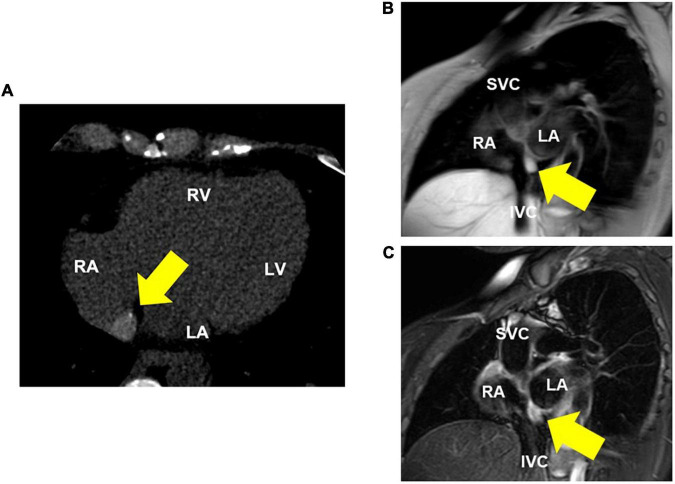
Thoracic computed tomography (CT) and MRI (T1 and T2-weighted) images. **(A)** CT shows a high-intensity nodule on the interatrial septum. **(B)** Cardiac MRI (T1-weighted image) reveals a nodule that is little higher signal than its surrounding muscle. **(C)** Cardiac MRI (T2-weighted image) reveals a high-intensity nodule on the interatrial septum, suggesting a cystic lesion with fluid components. RA, right atrium; RV, right ventricle; LA, left atrium; LV, left ventricle; SVC, superior vena cava; IVC, inferior vena cava.

Transesophageal echocardiography revealed a 16 × 10 mm isoechoic mass lesion on the right interatrial septum. The mass had a clear border, smooth surface, and broad base without mobility and apparent feeding vessels (Figure 1; Supplementary Videos 1, 2). A plain thoracic computed tomography (CT) revealed a high-intensity nodule on the right interatrial septum at the border of the inferior vena cava (Figure 2A). The absorption value was 110 HU and size was 13 × 9 mm. Moreover, there was no change in absorption value before and after contrast enhancement. Cardiac MRI showed a 14-mm nodule attached to the inter atrial septum. The nodule showed little higher signal than its surrounding muscle on a T1-weighted image. In addition, the tumor had high signal intensity on a T2-weighted image. No significant abnormal enhancement in LV wall and nodule on a late gadolinium enhancement image. Based on these findings, the nodule was suggested to be a cystic mass including liquid component (Figures 2B,C). In addition, whole-body positron emission tomography-CT showed no fluorodeoxyglucose accumulation in the lesion.

**FIGURE 3:**
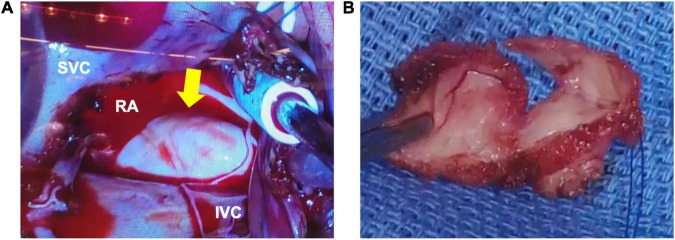
Macroscopic findings during surgery. **(A)** The outer surface of the mass is white, smooth, and soft. **(B)** A yellowish-white viscous mucous material is found in the mass. There are no thrombi inside or outside the cyst. RA, right atrium; RV, right ventricle; LA, left atrium; LV, left ventricle; SVC, superior vena cava; IVC, inferior vena cava.

**FIGURE 4:**
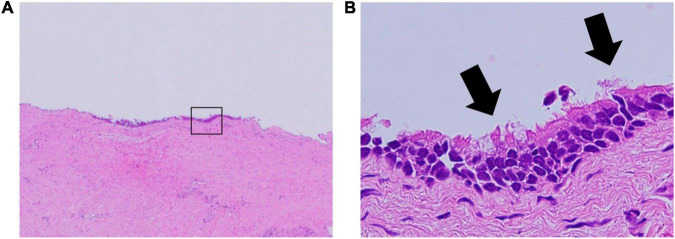
Pathology of the bronchogenic cyst. **(A)** Hematoxylin and eosin stain; original magnification, 20×. **(B)** The ciliated columnar epithelium suggests a bronchogenic cyst; original magnification, 400×.

The right atrial cyst was resected using minimally invasive cardiac surgery *via* right mini-thoracotomy. After aortic clamping, induction of cardiac arrest, and incision of the right atrium, a white mass with a smooth surface appeared on the posterior wall of the right atrium-inferior vena cava junction. A yellowish-white viscous mucous material was found in the mass with no thrombus (Figure 3). The mass was completely resected, and the right atrial posterior wall defect in the inferior vena cava was reconstructed using a bovine pericardial patch. Pathohistological evaluation revealed multilineage ciliated columnar epithelium and mucous glands present, resembling the respiratory epithelium in the intima of some cyst walls (Figure 4). No findings, such as nuclear irregularities or atypical cells, suggested malignancy. Therefore, the patient was diagnosed with a bronchogenic cyst (11).

The patient’s postoperative course was favorable and uneventful. She was discharged 21 days later without any complications. Currently, the patient visits the hospital as an outpatient without recurrence.

## Discussion

This was an extremely rare case of bronchogenic cyst occurring in a right atrium, in which establishing an accurate preoperative diagnosis was difficult. The differential diagnoses of intra-cardiac tumors include myxomas, lipomas, papillary fibroelastomas, metastatic tumors, cardiac cysts, or thrombi. Myxomas are the most frequent benign cardiac tumors occurring in adulthood: asymptomatic in 0–20% of cases (12). Lipomas are adipocyte-derived masses occurring in the epicardial myocardium, with approximately 25% of these lesions occur in the intracardiac lumen (13). These tumors are generally observed as isoechoic masses when the capillary blood flow in the stalk becomes obstructed, the mass becomes necrotic, and its echo-image is hypoechogenic (14). Papillary fibroelastomas generally occur on the valvular surface and are most commonly found in the aortic valve (15). In this present case, tumor markers were not elevated, leading to ruling out a metastatic cardiac tumor. The D-dimer level also indicated a low likelihood of thrombosis. Pericardial cysts are rare benign disease that often identified incidentally on chest X-ray or trans-thoracic echocardiography, the majority of cases of this disease are asymptomatic. Since complex type pericardial cysts are defined as the presence of solid compornents, it was difficult to distinguish between pericardial cysts and this case. However, these diseases are more likely to be located at the right cardiophrenic angle followed by the left cardiophrenic angle in mediastinal sites (16). Thus, we ruled out pericardial cysts. Although, differential diagnosis as an extra-cardiac tumors include substernal thyroid, thymic tumor, intra-thoracic cystic hygroma, serous cyst, and aortic aneurysm, all they were excluded by multimodality imaging studies. In this case, multimodality imaging suggested that the cystic lesion contained both fluid and blood. Thus, the preoperative clinical diagnosis was suspected to be a right atrial cyst.

Cardiovascular magnetic resonance (CMR) imaging is an important diagnostic tool for evaluating patients with suspected cardiac tumors. A previous study demonstrated that CMR diagnoses were 25% no mass, 16% pseudo mass, 16% thrombus, 17% benign tumor, and 23% malignant tumor (17). Compared to the final diagnosis, the CMR diagnosis was accurate in 98.4% of patients; patients with CMR diagnoses of pseudo mass and the benign tumor had mortality rates similar to those without masses, but patients with malignancy [hazard ratio (HR) 3.31 (2.40–4.57)] and thrombus [HR 1.46 (1.00–2.11) (17). The CMR diagnosis had more prognostic value than clinical factors such as left ventricular ejection fraction, coronary artery disease, or history of extracardiac malignancy (*P* < 0.001) (17). Therefore, we should perform CMR for evaluating patients with suspected cardiac tumors since CMR diagnosis is a powerful independent predictor of mortality over clinical risk factors (18).

We reviewed all case reports of recent intra-cardiac bronchogenic cyst published and summarized them in Table 2. Common symptoms of bronchogenic cysts include cough, fever, and dyspnea (19). Miwa et al. reported a case of a bronchogenic cyst in the lower interatrial septum that demonstrated complete atrioventricular block (20). In this case, the patient had paroxysmal atrial fibrillation and a cyst in the right interatrial septum near the inferior vena cava. Although the causal relationship between tumors and arrhythmias is not clear, arrhythmia of unknown origin in younger patients may lead clinicians to suspect a cardiac tumor. Furthermore, in some cases, obstruction of the superior vena cava (SVC) and dysphagia have been reported (21, 22). In addition, bronchogenic cysts can cause compression into the left atrium, pulmonary veins, or coronary arteries (23, 24). Regarding these reasons, it might be essential to focus on the anatomical location of the cyst accurately.

**TABLE 2:** Literature review of recent intra-cardiac bronchogenic cysts.

No	References	Age	Sex	Location	Symptoms	Arrythmia	Treatment
1	Foley (28)	28	F	IAS	Incidentally	No	Conservative
2	Shiferaw (29)	42	M	Myocardium	Chest pain, fatigue	No	Unrelated sudden death
3	Hui (9)	36	F	IAS	Palpitation	No	Resection
4		29	F	IAS	Dyspnea	No	Resection
5	Olsen (30)	50	F	IAS	Cough, dyspnea	No	Resection
6	Forcillo (31)	41	F	IVS	No, heart murmur	No	Resection
7	Grozavu (32)	42	F	Pericardium	Dull chest pain	No	Resection
8	Wang (33)	41	M	LV wall	precordial pain	No	Resection
9	Smer (34)	50	M	IAS	Tachycardia	Af	Resection
10	Shiohira (35)	77	F	AVS	Syncope	Wenckebach AVB, Vf	Resection
11	Nishida (36)	78	M	IVS	Unknown	Af, PVC	Unrelated death
12	Eriko (20)	36	M	IAS	Chest discomfort	Third-degree AVB	Resection and PM
13	Blesneac (37)	10	F	IVS	Incidentally	PVC, PAC	Resection
14	Van Praet (38)	58	M	IAS	Stroke	No	Resection
15	Gimpel (39)	71	F	Pericardium	Dysphagia, SOB	No	Resection
16	Li (40)	17	M	LA, RSPV	Chest pain, dyspnea	No	Resection
17	Fukada (41)	42	F	IAS	Dyspnea on exertion	No	Resection
18	Essam (42)	31	F	IAS	Retrosternal pain	Tachycardia, atrial flutter	Resection
19	Luo (10)	47	M	IAS	SOB	No	Resection

*M, male; F, female; LV, left ventricle; LA, left atrium; IAS, Inter atrial septum; IVS, Inter ventricular septum; AVS, atrio-ventricular septum; RSPV, right superior pulmonary vein; AVB, atrioventricular block; Af, atrial fibrillation; Vf, ventricular fibrillation; PVC, Premature Ventricular Contraction; PAC, premature atrial contraction; AF, atrial flutter; SOB, shortness of breath; PM, pacemaker.*

Whether to perform a surgery or biopsy first is a dilemma in diagnosing rare cardiac tumors or cysts. Puncture aspiration biopsy was an option for preoperative diagnosis. However, biopsy of a cystic lesion could rupture the trachea, thoracic cavity, or pericardial cavity. In addition, there has been a reported case of cystic infection after endobronchial ultrasonography-guided fine needle aspiration. Therefore, cystic lesions in the intra-cardiac cavity should not be considered for a needle aspiration (25). Given these complications, direct surgical resection is recommended without prior tumor biopsy (26). In addition, bronchogenic cyst is a risk of malignant transformation in adulthood, and risk of complications increase over time. Therefore, bronchogenic cysts are recommended for preferably early surgery (27). Since it could not be judged whether the tumor was benign or malignant, we decided to resect it directly. It must be emphasized that preoperative diagnosis can also be complicated by the extreme rarity of such cases. This case describes the usefulness of multimodality imaging in diagnosing bronchogenic cysts preoperatively with minimal complications.

## Conclusion

There is a clinical dilemma surrounding the treatment plan for cardiac surgery or biopsy of cardiac masses, especially in patients with rare cardiac cysts. The anatomical location of the cyst is related to various clinical symptoms and complications. Therefore, multimodality imaging might be practical for assessing the exact anatomical location of cysts and avoiding various complications. This case describes a rare bronchogenic cyst in the right atrium and highlights the importance of multimodality imaging for appropriate diagnosis and management. In cases of indeterminate cardiac cysts, direct removal of the cyst without previous biopsy is of utmost importance.

## Data Availability Statement

The raw data supporting the conclusions of this article will be made available by the authors, without undue reservation.

## Ethics Statement

Ethical review and approval was not required for this study on human participants in accordance with the local legislation and institutional requirements. The patients/participants provided their written informed consent to participate in this study.

## Author Contributions

YF and MH contributed significantly to the writing and editing to the manuscript. MH, SMa, TY, SF, KI, SMo, and MF managed the patient. TU and AS performed the surgeries. All authors critically revised the report, commented on the drafts of the manuscript, and approved the final version for publication.

## Conflict of Interest

The authors declare that the research was conducted in the absence of any commercial or financial relationships that could be construed as a potential conflict of interest.

## Publisher’s Note

All claims expressed in this article are solely those of the authors and do not necessarily represent those of their affiliated organizations, or those of the publisher, the editors and the reviewers. Any product that may be evaluated in this article, or claim that may be made by its manufacturer, is not guaranteed or endorsed by the publisher.
